# Association between Progressive Intraventricular Conduction Disturbance and Cardiovascular Events

**DOI:** 10.1371/journal.pone.0157412

**Published:** 2016-07-08

**Authors:** Hideki Hayashi, Qi Wu, Minoru Horie

**Affiliations:** Department of Cardiovascular and Respiratory Medicine, Shiga University of Medical Science, Otsu, Japan; Osaka University Graduate School of Medicine, JAPAN

## Abstract

**Background:**

Prolonged QRS duration on electrocardiogram (ECG) has been known as a poor prognostic marker. However, little is known about association between progressive intraventricular conduction disturbance and cardiovascular prognosis.

**Methods:**

From among a database containing 359,737 12-lead ECG recordings, patients whose QRS duration progressively increased from <120 msec to ≥120 msec were selected using software. The prognosis of patients was searched by medical record. The primary endpoint was defined as heart failure hospitalization. The secondary endpoint was heart failure hospitalization, device implantation, or cardiovascular death.

**Results:**

A total of 143 patients (100 males; age, 58.9±11.1 years) were enrolled in this study. QRS duration increased by 46.4±13.8 msec, manifesting right bundle branch block (RBBB) in 99 (69.2%) patients and non-RBBB (i.e., left bundle branch block, RBBB with left anterior hemiblock, or nonspecific intraventricular conduction disturbance) in 44 (30.8%). During the follow-up (mean, 16.6±5.3 years), 44 (30.3%), 15 (10.3%), and 6 (4.1%) patients resulted in heart failure hospitalization, device implantation, and cardiovascular death, respectively. Multivariate Cox proportional hazards models revealed that 1) the temporal increase in QRS duration was associated with the primary endpoint (hazard ratio [HR] 1.98; 95% confidence interval [CI] 1.05–3.80; p = 0.04) and the secondary endpoint (HR 2.79; 95% CI 1.55–5.00; p = 0.0001) and 2) the development of non-RBBB was associated with the primary endpoint (HR 3.02; 95% CI 1.59–5.73; p = 0.0001) and the secondary endpoint (HR 2.82; 95% CI 1.57–5.09; p = 0.001).

**Conclusion:**

The temporal increase in QRS duration and the development of non-RBBB patterns were independently associated with adverse cardiovascular prognosis.

## Introduction

QRS duration on a standard 12-lead electrocardiogram (ECG) has been considered an important prognostic marker in individuals with or without structural heart disease. It was reported that prolonged QRS duration was associated with increased risk of cardiovascular mortality[1] and reduced left ventricular ejection function.[2] In addition, QRS morphology has been taken into account in terms of a prognostic factor of cardiovascular disease. In general, right bundle branch block (RBBB) has been considered benign;[3] in contrast, left bundle branch block (LBBB) has been associated with adverse prognosis.[4] Recently, non-specific intraventricular conduction disturbance (NSIVCD) yielded higher risk of cardiac death and arrhythmic death in general population.[5] Accordingly, both QRS duration and QRS morphology can provide critical information on cardiovascular prognosis.

Despite the importance of QRS complex, little is known about prognostic value of progressively developed intraventricular conduction disturbance. Therefore, we sought to examine the association of progressively developed intraventricular conduction disturbance with cardiovascular events in a hospital-based population using a large database.

## Subjects and Methods

### Study Population

In the University Hospital of Shiga University of Medical Science, digital recording of 12-lead ECG started from January 1983. Details of the ECG database and the data collection were described previously.[6] From the database, we chose patients whose QRS duration progressively increased from <120 msec to ≥120 msec enrolled in this study. These patients consisted of part of our previous study.[6] To determine the temporal increase in QRS duration, the time from the first ECG recording to the last one was at least ≥1 year. ECGs exhibiting Wolff-Parkinson-White syndrome, ventricular pacing, junctional or idioventricular rhythm and ventricular tachyarrhythmias were excluded. Patients who were <15 years old or had abnormal QRS morphologies in the baseline ECG recording were also excluded from the analysis. The research protocol was approved by the Institutional Review Board of Shiga University of Medical Science (approval number: 19–75). Medical record was anonymized prior to analysis.

### ECG Analysis

The ECG analysis was performed using software (MUSE7.1, GE Marquette Medical Systems, Inc., Milwaukee, Wisconsin). Standardized, computerized ECG criteria as described by a 12-lead ECG analysis program were used to diagnose ventricular conduction morphologies such as RBBB, LBBB, left anterior hemiblock (LAH), and NSIVCD. The QRS morphology was double-checked by experienced cardiologists. The methods of ECG measurement were described in detail previously.[6] Because all variables of 12-lead ECGs were digitally measured based on computer analysis, neither intra-observer nor inter-observer variability should be taken into account.

### Follow-up

The follow-up period of all patients started from the time when an ECG with QRS duration being <120 msec was taken. We explored the prognostic factors for the end points of this study: heart failure hospitalization and cardiovascular events (i.e., cardiovascular death, heart failure hospitalization, or device implantation). The outcome was assessed by searching medical records stored in the University Hospital. Heart failure hospitalization had to satisfy both of the following criteria: 1) admission to hospital for ≥ 24 hours with a clinical history of worsening symptoms of heart failure as evidenced by clinical criteria, including increased New York Heart Association functional class, orthopnea, paroxysmal nocturnal dyspnea, edema, exertional dyspnea, or gastrointestinal symptoms attributable to heart failure, and 2) one or more intensive treatments for heart failure within 24 hours of admission, such as intravenous diuretics or inotropic agents. Device therapy included the implantation of cardiac pacemaker, implantable cardioverter-defibrillator (ICD), and cardiac resynchronization therapy (CRT) device. All ECGs taken during the follow-up period were evaluated in all patients enrolled in this study. An ECG that was taken for the first time was assigned as a baseline ECG, and an ECG recording when the end points occurred during the follow-up period was assigned as a follow-up ECG. Death due to cardiac causes was determined from the relevant International Classification of Diseases codes. To identify cases of sudden death due to arrhythmia, all deaths due to cardiac causes were reviewed by experienced cardiologists.

### Statistical Analysis

We described continuous variables using mean and standard deviation (SD), and categorical variables using number and percentage. Comparisons between groups were made by *t*-test for continuous variables and χ^2^-square test for categorical variables. Receiver operating characteristic curve was used to determine a cutoff point of prognostic factors to optimize sensitivity and specificity of ECG variables for endpoints. The Kaplan-Meier curve was made to describe event-free survival rate and the difference between groups were compared by log-rank test. Multivariate Cox proportional-hazards models were used to estimate hazard ratios of heart failure hospitalization and cardiovascular events (i.e., cardiovascular death, heart failure hospitalization, or device implantation) after adjusting age, gender, and other factors. Variables included in the Cox model were selected by a variable procedure with a criteria of p<0.1 for inclusion. All tests were two-tailed and a significant level was set in 0.05.

## Results

### Clinical Characteristics of the Patients

A total of 143 consecutive patients (100 men; mean age, 58.9±11.1 years) who met the inclusion criteria were selected from among database using software. The baseline clinical characteristics of the enrolled patients are listed in Table 1. The study population consisted of patients with various cardiac diseases and other disorders including diabetes mellitus and dyslipidemia. The mean duration of follow-up was 16.6±5.3 years. The number of ECG recordings averaged 17.8±21 (median: 11) a patient. The heart rate did not significantly change during the follow-up (baseline ECG, 69.9±12.9 beats/min vs. follow-up ECG, 67.7±14.7 beats/min; p = 0.20). The QRS duration increased from 93.6±7.5 ms (baseline ECG) to 140.0±12.5 ms (follow-up ECG). During the follow-up, RBBB, RBBB with LAH, LBBB, and NSIVCD patterns were developed in 99 (69.2%) patients, 9 (6.3%), 22 (15.4%), and 13 (9.1%), respectively.

**Table 1:** Baseline clinical characteristics of the study population (n = 143).

Age (years)	58.9 ± 11.1
Male—n (%)	100 (69.9)
Follow-up period (years)	16.6 ± 5.3
Organic heart disease* - n (%)	21 (14.7)
Hypertension—n (%)	47 (32.9)
Diabetes mellitus—n (%)	33 (23.1)
Dyslipidemia—n (%)	13 (9.1)

Data are presented as mean ± SD or n (%). The follow-up period is a time interval from the baseline ECG recording to the endpoint or the last day of observation.

*Organic heart disease indicates ischemic heart disease, cardiomyopathy, valvular heart disease, and congenital heart disease.

### Long-term Prognosis

Among the enrolled patients in this study, 44 (30.8%) patients were hospitalized because of heart failure, and 15 (10.5%) underwent device implantation. Pacemaker, ICD, and cardiac resynchronization therapy with defibrillator (CRT-D) were implanted in 13, 1, and 1 patients, respectively. Pacemaker was implanted for atrioventricular block in 10 patients and sick sinus syndrome in 3 patients. ICD was implanted for ventricular tachycardia occurred in hypertrophic cardiomyopathy. CRT-D was applied for heart failure with ventricular tachycardia and heart failure occurred in ischemic cardiomyopathy. In addition, 20 patients died, in which 6 (4.2%) patients suffered from cardiovascular death.

### Heart Failure

At baseline, the age was not significantly different between patients with and without heart failure hospitalization (61.3±11.2 years vs. 57.8±11.6 years, p = 0.0798), and the male prevalence was not significantly different between patients with and without heart failure hospitalization (73.3% vs. 68.4%, p = 0.5450). The follow-up period was not significantly different between patients with and without heart failure hospitalization (16.4±5.4 years vs. 16.7±5.2 years, p = 0.8239). Table 2 shows ECG characteristics according to the presence or absence of heart failure hospitalization. In the baseline ECG, the heart rate was not significantly different between the two groups, and the QRS duration was not significantly different between the two groups. In the follow-up ECG, the heart rate was not significantly different between the two groups, but the QRS duration was marginally significantly longer in patients with heart failure hospitalization than in those without. The temporal increase in QRS duration was significantly larger in patients with heart failure hospitalization than in those without. The prevalence of an RBBB pattern was significantly lower in patients with heart failure hospitalization than in those without. In contrast, the prevalence of a LBBB pattern was significantly higher in patients with heart failure hospitalization than in those without. The prevalence of a NSIVCD pattern tended to be higher in patients with heart failure hospitalization than in those without. The prevalence of an RBBB pattern with LAH was identical between patients with and without heart failure hospitalization.

**Table 2:** Comparison of ECG characteristics according to heart failure hospitalization.

	Heart failure hospitalization (+) (n = 44)	Heart failure hospitalization (-) (n = 99)	p value
**Baseline ECG**			
Heart rate (beats/min)	70.6±12.2	69.6±13.2	0.6528
QRS duration (ms)	92.7±6.9	93.9±7.8	0.3465
**Follow-up ECG**			
Heart rate (beats/min)	67.4±18.4	67.8±12.8	0.8968
QRS duration (ms)	142.8±14.3	138.6±11.5	0.0690
Temporal increase in QRS duration (ms/year)*	6.4±7.0	4.4±2.6	0.0184
RBBB pattern (n, %)	21, 46.7	78, 79.6	<0.0001
RBBB pattern with LAH (n, %)	3, 6.7	6, 6.1	0.9014
LBBB pattern (n, %)	14, 31.1	8, 8.2	0.0007
NSIVCD pattern (n, %)	7, 15.6	6, 6.1	0.0792

Data are presented as mean ± SD or n (%). RBBB = right bundle branch block, LAH = left anterior hemiblock, LBBB = left bundle branch block, NSIVCD = nonspecific intraventricular conduction disturbance.

*Temporal increase indicates the difference in measures of QRS duration between the baseline ECG and the follow-up ECG.

**Fig 1:**
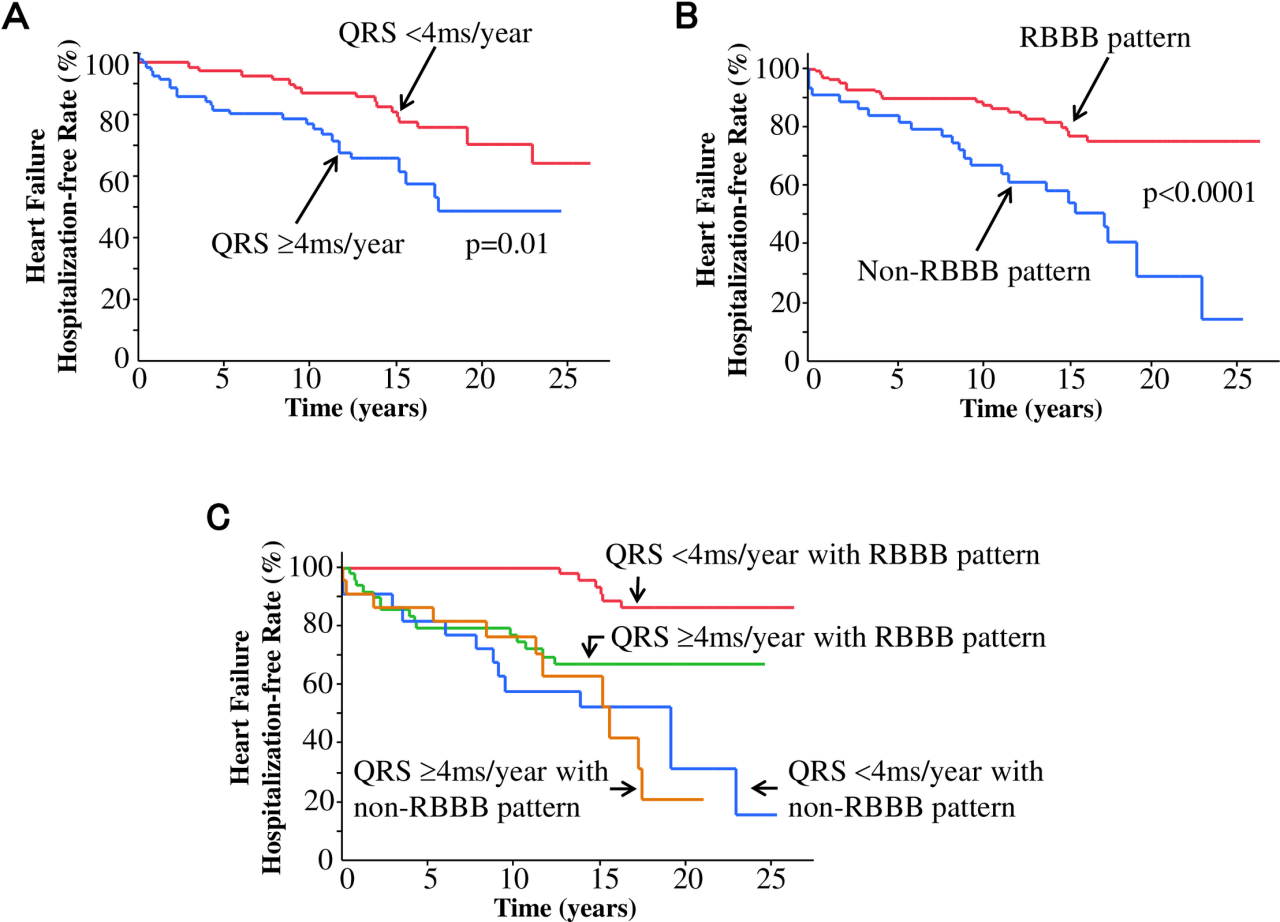
Kaplan-Meier survival analysis for hospitalization for heart failure. (A) Temporal increase in QRS duration of <4ms/year (red line) versus ≥4 ms/year (blue line). (B) Development of a right bundle branch block (RBBB) pattern (red line) versus a non-RBBB [i.e., left bundle branch block (LBBB), RBBB with left anterior hemiblock, or non-specific intraventricular conduction disturbance (NSIVCD)] pattern (blue line). (C) Classification into four groups based on the temporal increase in QRS duration and the development of BBB patterns: patients with the temporal increase in QRS duration of <4 ms/year and the development of an RBBB pattern (red line), patients with the temporal increase in QRS duration of ≥4 ms/year and the development of an RBBB pattern (green line), patients with the temporal increase in QRS duration of <4 ms/year and the development of a non-RBBB pattern (blue line), and patients with the temporal increase in QRS duration of ≥4 ms/year and e development of a non-RBBB pattern.

The cumulative incidence of heart failure hospitalization is shown in Fig 1. The temporal increase in QRS duration of ≥4 ms/year was associated with a significantly increased risk of heart failure hospitalization than that of <4 ms/year (hazard ratio [HR], 2.18; 95% confidence interval [CI], 1.20–4.07; p = 0.01) (Fig 1A). The development of a non-RBBB pattern (i.e., RBBB with LAH, LBBB, or NSIVCD pattern) was associated with a significantly increased risk of heart failure hospitalization than that of an RBBB pattern (HR, 3.32; 95% CI, 1.84–6.04; p<0.0001) (Fig 1B). Table 3 shows univariate and multivariate survival analyses in association with heart failure hospitalization. In multivariate analysis, after adjustment for confounding factors, heart failure hospitalization was independently associated with the temporal increase in QRS duration ≥4 ms/year (HR, 1.98; 95% CI, 1.05–3.80; p = 0.04) and the development of a non-RBBB pattern (HR, 3.02; 95% CI, 1.59–5.73; p = 0.0001). Because both temporal increase in QRS duration and developed QRS morphology were independently associated with heart failure hospitalization, patients were classified into four groups: 1) patients with the QRS duration of <4 ms/year with the development of an RBBB pattern, 2) patients with the QRS duration of ≥4 ms/year with the development of an RBBB pattern, 3) patients with the QRS duration of <4 ms/year with the development of a non-RBBB pattern, and 4) patients with the QRS duration of ≥4 ms/year with the development of a non-RBBB pattern. Fig 1C shows the cumulative incidence of heart failure hospitalization of these four groups. Compared to patients with the temporal increase in QRS duration of <4 ms/year with the development of an RBBB pattern, patients with the temporal increase in QRS duration of ≥4 ms/year with the development of an RBBB pattern had a significantly increased risk of heart failure hospitalization (HR, 4.53; 95% CI, 1.82–12.84 p = 0.001) after adjustment for confounding factors. Patients who developed a non-RBBB pattern had much worse prognosis with the temporal increase in QRS duration being either <4 ms/year (HR, 7.52; 95% CI, 2.96–21.45; p<0.0001) or ≥4 ms/year (HR, 7.73; 95% CI, 2.90–22.74; p<0.0001) after adjustment for confounding factors, compared to patients with the temporal increase in QRS duration of <4 ms/year with the development of an RBBB pattern.

**Table 3:** Univariate and multivariate survival analyses of heart failure hospitalization.

Variables	Univariate analysis		Multivariate analysis*	
	p value	HR (95% CI)	p value	HR (95% CI)
Age (60 years old = 1)	0.12	1.60 (0.88–3.01)	0.63	1.18 (0.61–2.35)
Gender (female = 1)	0.49	1.26 (0.67–2.54)	0.72	0.87 (0.42–1.90)
Temporal increase in QRS duration (4 ms/year = 1)	0.01	2.18 (1.20–4.07)	0.04	1.98 (1.05–3.80)
Non-RBBB pattern (RBBB pattern = 1)	<0.0001	3.32 (1.84–6.04)	0.0001	3.02 (1.59–5.73)

*Adjusted for age, sex, heart rate, intraventricular conduction pattern (i.e., RBBB pattern vs. non-RBBB pattern), temporal increase in QRS duration, and presence of organic heart disease.

HR = hazard ratio, CI = confidence interval, RBBB = right bundle branch block.

### Cardiovascular Events

During the follow-up period, 53 patients developed cardiovascular events including heart failure hospitalization, device implantation, and cardiovascular death. At baseline, the age was not significantly different between patients with and without cardiovascular events (61.1±11.0 years vs. 57.7±11.1 years, p = 0.0790), and the gender prevalence was not significantly different between patients with and without cardiovascular events (73.6% of male vs. 67.8% of male, p = 0.4621). The follow-up period was not significantly different between patients with and without cardiovascular events (16.5±5.6 years vs. 16.6±5.1 years, p = 0.9080). Table 4 shows ECG characteristics according to the presence or absence of cardiovascular events. In the baseline ECG, the heart rate was not significantly different between the two groups, and the QRS duration was not significantly different between the two groups. In the follow-up ECG, the heart rate was not significantly different between the two groups, but the QRS duration was significantly longer in patients with cardiovascular events than in those without. The temporal increase in QRS duration was significantly larger in patients with cardiovascular events than in those without. The prevalence of a RBBB pattern was significantly lower in patients with cardiovascular events than in those without. In contrast, the prevalence of a LBBB pattern was significantly higher in patients with cardiovascular events than in those without. The prevalence of an RBBB pattern with LAH tended to be higher in patients with cardiovascular events than in those without. The prevalence of a NSIVCD pattern was not significantly different between patients with and without cardiovascular events.

**Table 4:** Comparison of ECG characteristics according to cardiovascular events.

	Cardiovascular events (+) (n = 53)	Cardiovascular events (-) (n = 90)	p value
**Baseline ECG**			
Heart rate (beats/min)	71.8±12.6	68.8±13.0	0.1838
QRS duration (ms)	92.8±7.4	94.0±7.6	0.3816
**Follow-up ECG**			
Heart rate (beats/min)	67.5±17.7	67.8±12.7	0.8818
QRS duration (ms)	142.7±14.1	138.3±11.3	0.0398
Temporal increase in QRS duration (ms/year)*	6.1±6.5	4.4±2.7	0.0310
RBBB pattern (n, %)	25, 47.2	74, 82.2	<0.0001
RBBB pattern with LAH (n, %)	6, 11.3	3, 3.3	0.0629
LBBB pattern (n, %)	15, 28.3	7, 7.8	0.0012
NSIVCD pattern (n, %)	7, 13.2	6, 6.7	0.1969

Data are presented as mean ± SD or n (%). RBBB = right bundle branch block, LAH = left anterior hemiblock, LBBB = left bundle branch block, NSIVCD = nonspecific intraventricular conduction disturbance.

*Temporal increase indicates the difference in measures of QRS duration between the baseline ECG and the follow-up ECG.

**Fig 2:**
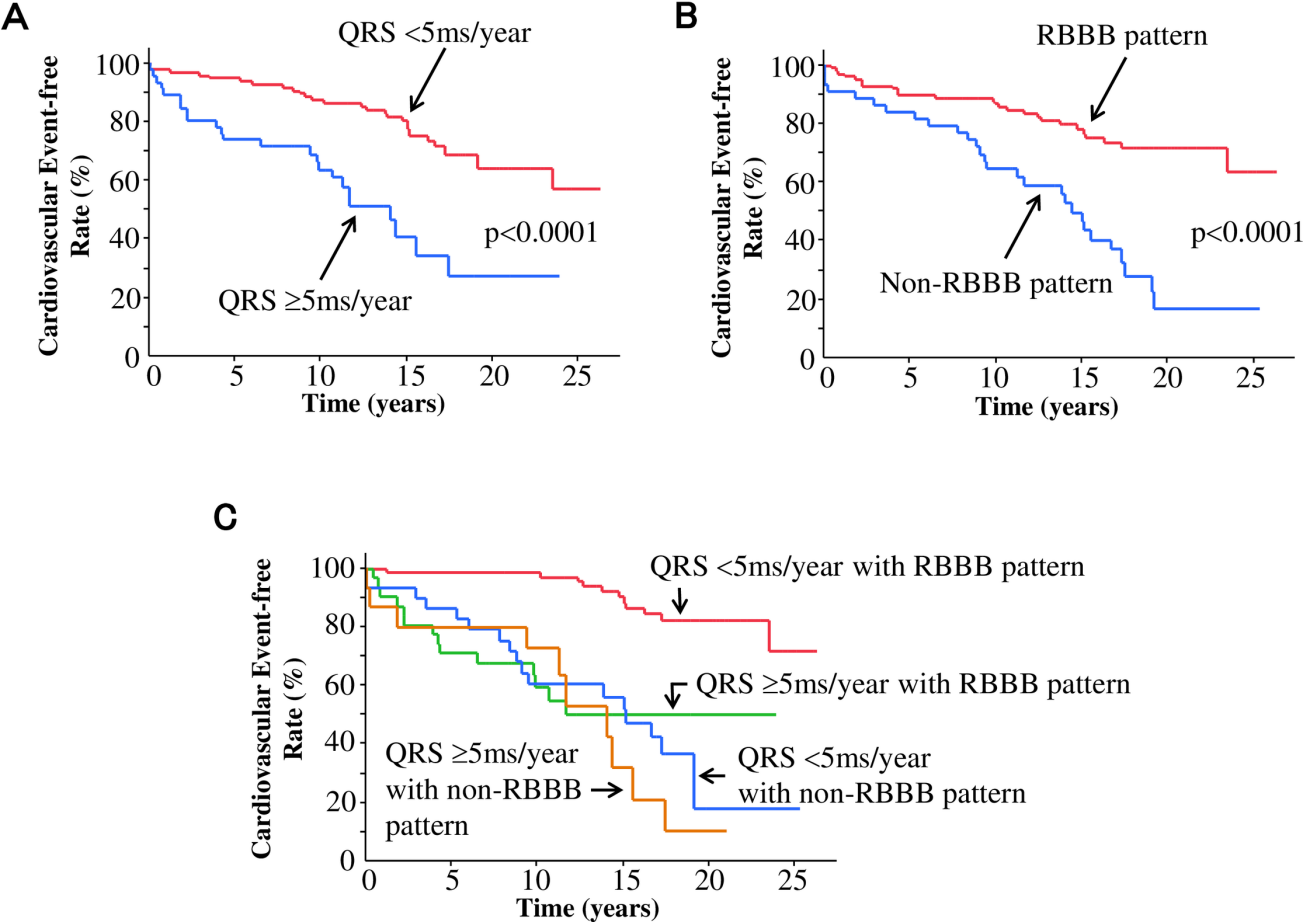
Kaplan-Meier survival analysis for cardiovascular events. (A) Temporal increase in QRS duration of <5 ms/year (red line) versus ≥5 ms/year (blue line). (B) Development of an RBBB pattern (red line) versus a non-RBBB (i.e., LBBB, RBBB with left anterior hemiblock, or NSIVCD) pattern (blue line). (C) Classification into four groups based on the temporal increase in QRS duration and the development of BBB patterns: patients with the temporal increase in QRS duration of <5 ms/year and the development of an RBBB pattern (red line), patients with the temporal increase in QRS duration of ≥5 ms/year and the development of an RBBB pattern (green line), the temporal increase in QRS duration of <5 ms/year and the development of a non-RBBB pattern (blue line), and patients with the temporal increase in QRS duration of ≥5 ms/year and the development of a non-RBBB pattern (orange line). Abbreviations are as Fig 1.

Fig 2 shows the cumulative incidence of cardiovascular events in association with QRS duration and QRS morphology. The temporal increase in QRS duration of ≥5 ms/year was associated with a significantly increased risk of cardiovascular events than that of <5 ms/year (HR, 3.33; 95% CI, 1.89–5.84; p<0.0001) (Fig 2A). The development of a non-RBBB pattern was associated with a significantly increased risk of cardiovascular events than that of an RBBB pattern (HR, 3.43; 95% CI, 1.99–5.94; p<0.0001) (Fig 2B). Table 5 shows univariate and multivariate survival analyses in association with cardiovascular events. The temporal increase in QRS duration and the development of a non-RBBB pattern were independently associated with cardiovascular events. According to the temporal increase in QRS duration and the developed QRS morphology, patients were classified into four groups: 1) patients with the QRS duration of <5 ms/year with the development of an RBBB pattern, 2) patients with the QRS duration of ≥5 ms/year with the development of an RBBB pattern, 3) patients with the QRS duration of <5 ms/year with the development of a non-RBBB pattern, and 4) patients with the QRS duration of ≥5 ms/year with the development of a non-RBBB pattern. Fig 2C shows the cumulative incidence of cardiovascular events in combination with QRS duration and QRS morphology. Compared to patients with the temporal increase in QRS duration of <5 ms/year with the development of an RBBB pattern, patients with the temporal increase in the QRS duration of ≥5 ms/year with the development of an RBBB pattern had a significantly increased risk of cardiovascular events (HR, 6.51; 95% CI, 2.90–14.95; p<0.0001) after adjustment for confounding factors. Patients who developed a non-RBBB pattern also had a significantly increased risk of cardiovascular events with the temporal increase in QRS duration being either <5 ms/year (HR, 6.08; 95% CI, 2.90–13.36; p<0.0001) or ≥5 ms/year (HR, 8.25; 95% CI, 3.39–19.85; p<0.0001) after adjustment for confounding factors, compared to patients with the temporal increase in QRS duration of <5 ms/year with the development of an RBBB pattern.

**Table 5:** Univariate and multivariate survival analyses of cardiovascular events.

Variables	Univariate analysis		Multivariate analysis*	
	p value	HR (95% CI)	p value	HR (95% CI)
Age (60 years old = 1)	0.09	1.61 (0.93–2.87)	0.92	1.03 (0.55–1.95)
Gender (female = 1)	0.43	1.27 (0.71–2.43)	0.65	0.85 (0.44–1.73)
Temporal increase in QRS duration (5 ms/year = 1)	<0.0001	3.33 (1.89–5.84)	0.0001	2.79 (1.55–5.00)
Non-RBBB pattern (RBBB pattern = 1)	<0.0001	3.43 (1.99–5.94)	0.001	2.82 (1.57–5.09)

Annotation and abbreviations are as Table 4.

*Adjustment for multivariate analysis is as Table 3.

## Discussion

Twelve-lead ECGs deserve not only a diagnostic tool but also a prognostic marker. To elucidate prognostic value of ECGs, a long-term follow-up is essential. A precise ECG analysis raises the study quality. We previously reported that the temporal increase in QRS duration from any QRS duration was associated with cardiovascular morbidity and mortality. In this study, we examined the long-term outcome in patients whose QRS duration progressively increased from <120 msec to ≥120 msec, with QRS morphology being investigated into the prognosis.[6] The major findings of this study are as follows: 1) the temporal increase in QRS duration was an independent prognostic predictor of heart failure hospitalization and cardiovascular events, 2) the development of a non-RBBB (i.e., LBBB, RBBB with left anterior hemiblock, or NSIVCD) pattern was an independent prognostic predictor of heart failure hospitalization and cardiovascular events, and 3) the temporal increase in QRS duration and the development of a non-RBBB pattern stratified the risk of heart failure hospitalization and cardiovascular events.

### QRS Duration and Prognosis

A number of studies reported that prolonged QRS duration was associated with adverse prognosis in cardiac disease,[2, 7–9] general population,[1, 10] and hospital-based population.[11] In addition, prolonged QRS duration was related to abnormal mechanical contraction of the left ventricle.[12, 13] Especially, LBBB is known as an ECG representation of adverse prognosis and decreased cardiac function, because LBBB causes dyssynchronous movement of the left ventricle.[14] These findings indicate that both QRS duration and QRS morphology matter in determining prognosis of cardiovascular disease. Recently, IVCD was reported to be associated with worse prognosis.[5] However, a prognostic study dealing with progressive intraventricular conduction disturbance is lacking. This study showed for the first time that not only the temporal increase in QRS duration but also the developed morphology of QRS complex was closely associated with adverse cardiovascular outcomes.

There are some reports about prognostic value of temporal increase in QRS duration. Regarding heart failure, QRS-duration lengthening of ≥0.5 msec/month was associated with a higher incidence of cardiac death or need for heart transplantation.[15] The other study reported that temporal increase in QRS duration was a predictor of heart failure deterioration in elderly patients.[16] These studies focused on QRS duration; contrary, our study evaluated the temporal increase in QRS duration and the developed morphology of QRS comples to determine the prognosis.

### QRS Morphology and Prognosis

In the Framingham study, prevalence of prolonged QRS duration increased proportionally with age of ≥40 years in both genders,^13^ and subjects with newly acquired BBB on ECG were associated with the presence or subsequent development of cardiovascular disease. In the same study, about 50% of patients with newly acquired LBBB developed coronary artery disease and heart failure.[17] In addition, the incidence of coronary artery disease and heart failure was higher in patients with newly acquired RBBB than in those without.[18]

A multicenter trial showed that LBBB and NSIVCD were significantly associated with increased risk of arrhythmic death and total mortality in patients with coronary artery disease.[9] But, this finding was not observed in patients with RBBB. Similarly, Aro et al.[5] reported an association of LBBB and NSIVCD with increased risk of arrhythmic death in a general population. Because the majority of development of abnormal intraventricular conduction was an RBBB pattern in this study, we compared patients with an RBBB pattern and those with a non-RBBB (i.e., RBBB with LAH, LBBB, or NSIVCD) pattern. This classification represents comparable sites where the abnormal intraventricular conduction pattern occurs: i.e., the right ventricle alone versus the left ventricle with or without the right ventricle. The present study showed for the first time that progressive intraventricular conduction disturbance excluding an RBBB pattern was associated with poor prognosis.

It has been reported that cardiac resynchronization therapy improved mortality and clinical symptoms in patients with heart failure.[19, 20] Patients with QRS prolongation of a LBBB pattern have reduced function of the left ventricle due to paradoxical septal motion, bulging of the lateral wall in early systolic phase, prolonging mitral regurgitation and reducing left ventricular filling time. Normalizing or decreasing QRS duration by cardiac resynchronization therapy improved hemodynamic performance.[21] These beneficial effects of cardiac resynchronization therapy may show the reverse process of the present study.

### Limitations

Because the retrospective cohort study was conducted using ECG database in the University Hospital, several limitations are inherent. First, we determined the increase of QRS duration by averaging temporal variation. The increase might not be temporally constant. To avoid the excess change during short time, we used the ECGs of which recoding interval was more than one year. Second, occurrence of intermittent BBB might have been missed unless the ECG finding was recorded in the hospital. Because no intermittent BBB could be documented during the follow-up in patients enrolled in this study, it is thought that intermittent BBB rarely occurred in this study population. Third, we did not involve cardiac function such as left ventricular ejection fraction and mitral regurgitation. It is speculated that left ventricular function might be temporally decreased proportionally with QRS prolongation, suggesting that QRS duration surrogates left ventricular ejection fraction. In fact, echocardiography was performed only in 17 of 143 patients (11.9%) at baseline. This small number prevented us from including left ventricular ejection fraction as one of covariates. Forth, the University Hospital plays a crucial and central role in treating patients around neighboring areas. In addition, no one failed to be followed in the present study. Therefore, it was very rare that patients who were treated in the University Hospital were transferred to other hospitals, when their health conditions became worse. Fifth, 20% of patients with the development of an RBBB pattern were hospitalized by heart failure due to myocardial infarction, hypertension, atrial fibrillation, valvular heart disease, or dilated cardiomyopathy. To elucidate the mechanistic interaction between the development of RBBB and the heart failure hospitalization, further investigation is needed. Lastly, because our study included patients who underwent ECG recording in our University Hospital, the risk of heart failure hospitalization in the study population was undoubtedly greater than that in general population. Therefore, this factor should be considered when our results are extrapolated to broader population.

### Conclusions

This study shows that progressive intraventricular conduction disturbance is associated with heart failure hospitalization and cardiovascular events. The temporal increase in QRS duration and the development of a non-RBBB pattern were independently associated with the adverse outcomes, thus serving as the risk stratification.
